# Multiscale Spectral Analysis on Lysozyme Aqueous Solutions in the Presence of PolyEthyleneGlycol

**DOI:** 10.3390/molecules27248760

**Published:** 2022-12-10

**Authors:** Maria Teresa Caccamo, Salvatore Magazù

**Affiliations:** 1 Dipartimento di Scienze Matematiche e Informatiche, Scienze Fisiche e Scienze Della Terra, Università degli Studi di Messina, Viale Ferdinando Stagno D’Alcontres 31, 98166 Messina, Italy; 2 Consorzio Interuniversitario Scienze Fisiche Applicate (CISFA), Viale Ferdinando Stagno d’Alcontres 31, 98166 Messina, Italy

**Keywords:** Lysozyme, PEG 400, thermal restraint, resilience

## Abstract

Infrared spectroscopy measurements were performed on Lysozyme aqueous solutions also in the presence of PolyEthylene Glycol (PEG 400) as a function of an increasing temperature from T = 27 °C to 90 °C, and, successively in sequence, by decreasing temperatures from T = 90 °C to 27 °C. Data were analyzed by evaluating the spectral difference with respect to the initial spectrum collected at 27 °C. This procedure allows to quantitatively evaluate the thermal restraint related to the thermal scan from T = 27 °C to 90 °C, as well as to introduce a spectral resilience concerning the entire increasing and decreasing thermal paths which allow to highlight the bioprotectant effectiveness of low molecular weight PEG. In particular, the main purpose of the present work is to highlight the effects of a thermal treatment on a mixture of Lysozyme/water and of Lysozyme/water/PEG 400 during an increasing temperature scan, and then after a successive decreasing temperature scan, in order to highlight the bioprotectant role of PEG 400. On that score, an evaluation of the spectral distances of the registered spectra as a function of increasing and decreasing temperatures has been performed and analyzed.

## 1. Introduction

It is well known that PolyEthylene Glycols (PEGs) are formed by repeating the unit of ethylene glycol, also called oxyethylene, whose chemical structure is H-(O-CH_2_-CH_2_)_m_-OH, *m* being the polymerization degree. They include both hydrophobic ethylene units (CH_2_-CH_2_) and hydrophilic oxygens. Due to the simplicity of their structure, this class of polymers is considered to be a good model for studying more complex systems, such as biostructures, proteins, and hydrophilic surfaces. For these reasons it has been investigated both from the theoretical and experimental point of view [1,2,3,4].

In particular, PEGs are cryo-protective for cells by limiting the increase in volume of water when it turns into ice; on the other hand, PEGs allow to lower the freezing temperature and, above certain concentrations, it is difficult to freeze the water contained in the capillaries. By way of example, for wood, PEG with Molecular weight M_w_ = 400, i.e., PEG 400, has the merits of combining good wettability, a powerful cryo-protective effect and good mechanical resistance to drying without the excessively shiny or sticky appearance that can have the lower M_w_ PEGs [5,6,7].

Even today, PEGs remain the most widely used products for the conservation of waterlogged organic materials such as wood, leather, and fibers. Their advantages are multiple: simultaneously consolidating, surfactant and cryo-protective, good penetration, ease of use, and safety for the operator. Their constraints and side effects—corrosive environment for metals, hygroscopicity, especially for low M_w_ PEGs, environment favorable to the development of microorganisms—are sufficiently well known and documented to adapt treatments, working and storage environments, as well as for monitoring tools [8,9,10,11].

In the present study we used PEG 400, which is a clear, simple, somewhat viscous, colorless, and odorless liquid. PEG 400 is one of the shortest polymers and corresponds to a chain length of 8 repetitive units. Its low toxicity and exceptional ability to solubilize polar active pharmaceutical ingredients make it highly valued in pharmaceutical applications [12,13,14,15,16,17]. PEG 400 is generally considered stable, non-reactive, soluble in all proportions with water, but also soluble in acetone, alcohols, benzene, glycerin, glycols, and aromatic hydrocarbons. This versatile excipient is used in topical, ophthalmic, oral, and parenteral pharmaceutical formulations [18,19,20,21,22].

In the present study, PEG 400 was added to aqueous solutions of a protein, i.e., Lysozyme, in order to investigate its effectiveness as bioprotectant during an increasing temperature ramp followed by a decreasing temperature ramp [23,24,25,26,27].

Lysozyme, discovered by Alexander Fleming in 1922, is a protein synthesized by white blood cells participating in the defense of the body through its proteolytic activity (it is a protease, protein degradation enzyme), which allows it to attack bacterial walls. In the form of a globular protein of 129 amino acids, Lysozyme is found in a certain number of secretions, in the secretions of granulocytes and monocytes, and in egg white [28,29,30,31,32].

The determination of the level of lysozyme in the blood, although very rarely practiced, makes it possible to control the effectiveness of the treatment of certain leukaemias; similarly, this measurement is useful in the diagnosis of certain kidney diseases or in monitoring organ transplant rejection [33,34,35,36,37].

**Figure 1:**
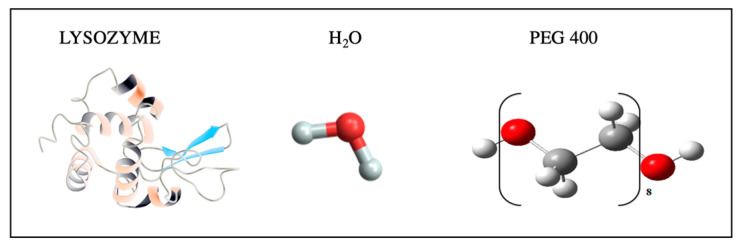
Molecular structures of H_2_O, PEG 400, and Lysozyme.

Figure 1 shows the molecular structures of the three solution components, i.e., H_2_O, PEG 400 and Lysozyme; concerning the PEG 400 structure it is important to highlight the hydrophilic character of PEG which interacts with water through its terminal groups, H and OH, and through its internal Oxygens.

The focus of our investigation is the characterization of the thermal response of these systems, and, on this purpose, the Infrared (IR) Spectroscopy technique has been employed. It useful to stress that molecular vibrations are what cause matter to absorb IR radiation, as molecular vibrational energy levels are separated by energies that fall into the infrared region of the electromagnetic spectrum. The infrared part of electromagnetic radiation is usually divided into three areas: the near infrared (the most energetic) which extends from 14.000 to 4.000 cm^−1^ (0.7–2.5 μm wavelength); the mid-infrared, which ranges from 4.000 to 400 cm^−1^ (2.5–25 μm); and, finally, the far-infrared, which covers the spectral range from 400 to 10 cm^−1^ (25–1000 μm). Due to its highly selective character, this spectroscopic technique is commonly used for the identification of compounds, but it also makes possible to obtain important information on inter- and/or intra-molecular interactions, on the conformation of molecules and on the organization of matter [38,39,40,41,42].

In order to analyze the thermal response of spectra obtained by means of IR technique an approach based on the Spectral Distance (SD) evaluation has been employed to compare spectra collected at different temperatures and hence to characterize sample changes with temperature.

Then, starting from the obtained SD values, we introduce the concept of spectral resilience for characterizing the ability of a material system to return to the initial state after suffering a thermal stress [43,44,45].

## 2. Results and Discussion

The investigation of the variations of the investigated spectra, by changing of temperature, has been performed by means of the Spectral Distance (SD) that is expressed by the following formula:

(1) $$SD=\sqrt{I\left(\omega ,T_{i}\right)-I\left(\omega ,T\right)}\;\cdot \;\Delta \omega$$

where I(ω,T) is the IR absorbance at frequency ω and at temperature T; $T_{i}$ is the initial or reference temperature; in our case, it is equal to 27 °C; and, finally, Δω represents the instrumental frequency resolution. Such an approach allows comparison of the profile of each spectrum for both the investigated systems, at different temperature values, with the reference spectra, taken at 27 °C [46,47].

**Figure 2:**
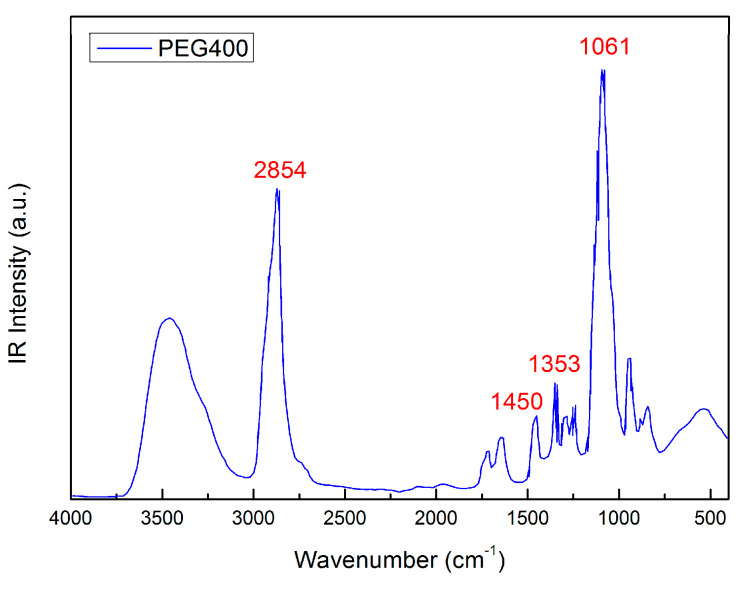
IR spectrum of PEG400 with band assignation; in particular, the peak at ~2869 cm^−1^ is attributed to -CH stretching, ~1456 cm^−1^ and ~1353 cm^−1^ is attributed to C-H bending, and ~1061 cm^−1^ is attributed to C-O stretching.

Figure 2 reports the IR spectrum of PEG400 with band assignation; in particular, the peak at ~2869 cm^−1^ is attributed to -CH stretching, ~1456 cm^−1^ and ~1353 cm^−1^ is attributed to C-H bending, and ~1061 cm^−1^ is attributed to C-O stretching [48,49].

**Figure 3:**
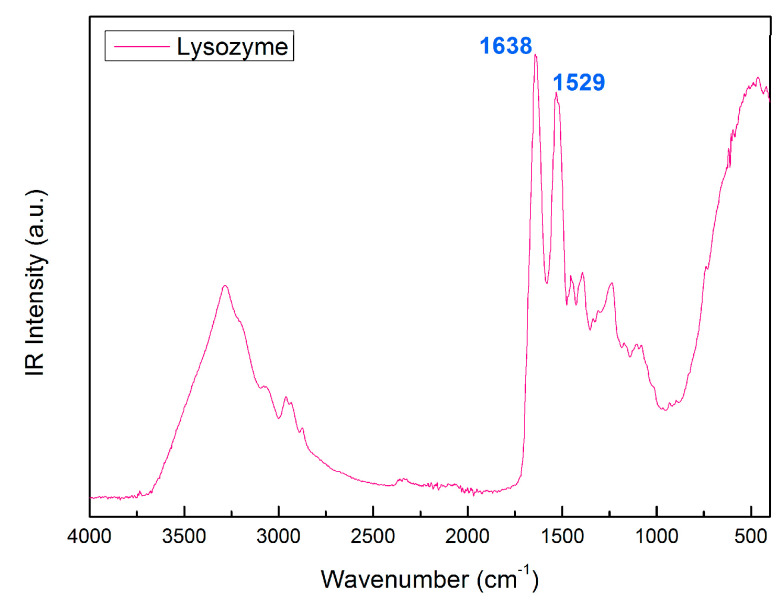
IR spectrum of Lysozyme with the assignation of Amide I and Amide II, at 1638 cm^−1^ and 1529 cm^−1^, respectively.

Figure 3 shows the IR spectrum of PEG 400 with the assignation of Amide I and Amide II, at 1638 cm^−1^, and 1529 cm^−1^, respectively [50,51,52].

**Figure 4:**
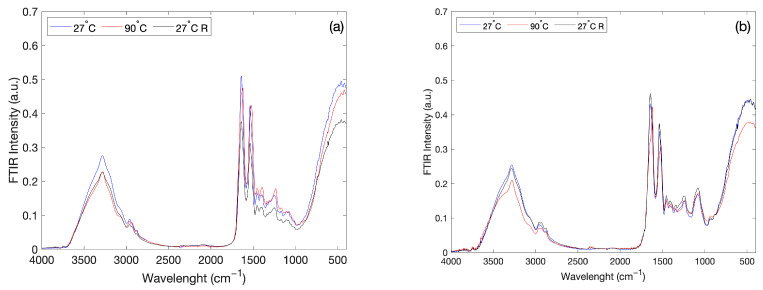
FTIR spectra of aqueous solution of Lysozyme (**a**) and FTIR spectra of aqueous solution of Lysozyme in presence of PEG400 (**b**) in the 400 < Δω < 4000 cm^−1^ spectral range at T = 27 °C, T = 90 °C and again at T = 27 °C, i.e., after that both the systems were brought to a temperature of 90 °C.

Figure 4a shows the FTIR spectra, in the 400 < Δω < 4000 cm^−1^ spectral range, of the aqueous solution of Lysozyme (binary system) at T = 27 °C, at T = 90 °C, i.e., at the lowest and highest values of the increasing temperature ramp, and again at T = 27 °C after that the system was brought to a temperature value of 90 °C; Figure 4b reports the same FTIR spectra for the aqueous solution of Lysozyme in presence of PEG 400 (ternary system).

As it can be seen, starting from the spectra collected at the lowest temperature value, i.e., T = 27 °C, after having heated both the binary and the ternary systems up to 90 °C, turning back to the temperature value of 27 °C, the spectra are different from the spectra initially acquired at 27 °C. On the other hand, the addition of PEG 400 to the aqueous solution of Lysozyme reduces the SD variations as a function of temperature.

It is useful to stress that Raman scattering on Lysozyme/water/bioprotectant systems have evidenced a similar behavior of the *Amide I* center frequencies [53].

In order to extract quantitative information on the system thermal response, we have evaluated the SD values for both the investigated systems taking into account the spectra collected within the increasing temperature ramp. The obtained SD values as a function of temperature fulfil an increasing sigmoid curve whose behavior is properly fitted by the following formula:

(2) $$SD\left(T\right)=A\left(1-\frac{1}{1+e^{B\left(T-T_{0}\right)}}\right)$$

where A represents the sigmoid amplitude, that is connected to the value of thermal restraint [41,50]; in particular, the thermal restraint value is the inverse of the sigmoid amplitude; *B* is the sigmoid steepness while $T_{0}$ is the relaxational temperature corresponding to the abscissa of the sigmoid inflection point [54,55].

**Figure 5:**
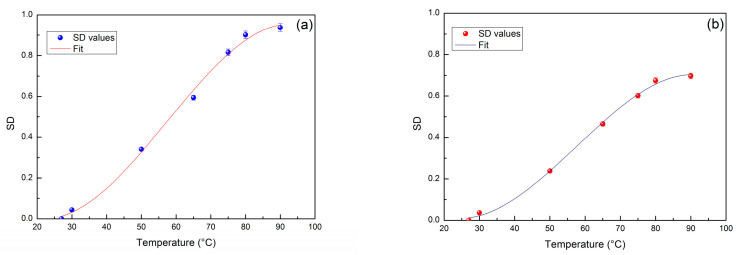
SD values, with error bars, as a function of temperature from 27 °C to  90 °C for (**a**) the binary system, i.e., Lysozyme/water, and (**b**) for the ternary system, i.e., Lysozyme/water/PEG 400. The obtained SD values as a function of temperature fulfil an increasing sigmoid behavior and have been fitted by means of Equation (2) which furnishes three parameter values, A, *B* and $T_{0}$.

Figure 5a reports the values of SD for the increasing temperature ramp, i.e., from 27 °C to 90 °C, for the binary system while Figure 5b reports the values of SD for the increasing temperature ramp for the ternary system; both the data are represented with their error bars.

What emerges from this analysis is that for the binary system, that value of amplitude A is 0.975 and the value of temperature $T_{0}$ is 55.32 °C. For the ternary system, the value of amplitude A is 0.697 and the value of temperature $T_{0}$ is 58.55 °C. Table 1 summarizes the values of A, B, and $T_{0}$ for the two investigated systems.

The obtained result shows that the addition of a small percentage of PEG 400 to Lysozyme gives rise to smaller changes in the features of the registered spectra, suggesting that the aqueous solution of Lysozyme in the presence of PEG 400 are more resistant to the temperature changes. 

Previous experiments performed by Raman scattering on Lysozyme/water/bioprotectant systems have evidenced a similar behavior for the protein *Amide I* center frequencies.

In order to characterize the thermal effects of our systems we have performed measurements not only following an increasing temperature ramp, but also during a further decreasing temperature ramp. On that score we introduce a new quantity that we call spectral thermal resilience, which is connected to the capability to recover the original spectral features after a thermal stress cycle. Therefore, the SD values for the two investigated systems have been calculated both for the increasing and the decreasing temperature ramps.

**Figure 6:**
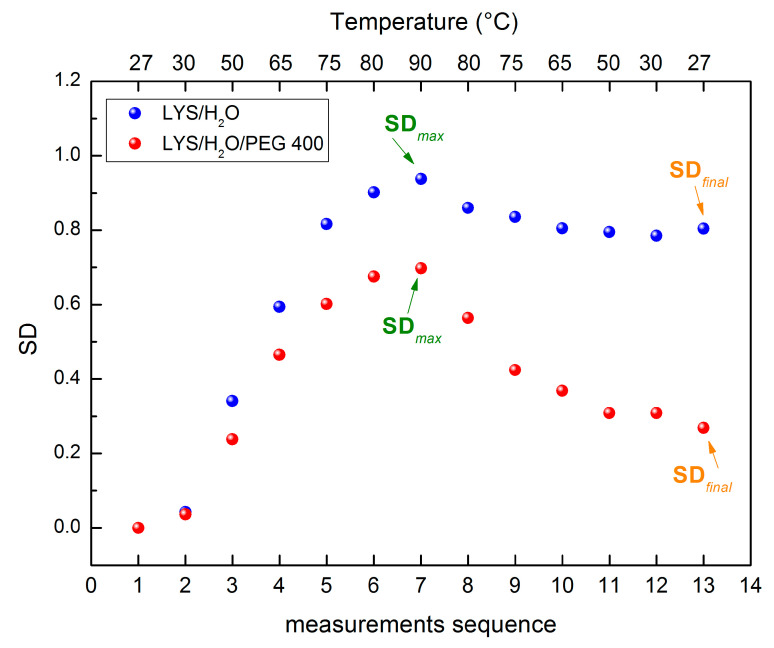
Calculated SD values during the full temperature cycle. The blue full circles refer to the SD values obtained for the Lysozyme aqueous solution while the magenta full circles refer to the SD values of Lysozyme aqueous solution in the presence of PEG 400. The label from 1 to 13 in the *y*-axes represents the sequence of the performed measurements, i.e., 1 = 27 °C, 2 = 30 °C, 3 = 50 °C, 4 = 65 °C, 5 = 75 °C, 6 = 80 °C, 7 = 90 °C, 8 = 80 °C, 9 = 70 °C, 10 = 65 °C, 11 = 50 °C, 12 = 30 °C, and 13 = 27 °C.

Figure 6 reports the behavior of the calculated SD values during the full temperature cycle. The blue full circles represent the SD values obtained for the Lysozyme aqueous solution while the magenta full circles represent the SD values of Lysozyme aqueous solution in the presence of PEG 400. The label from 1 to 13 in the y-axes represents the sequence of the performed measurements from 27 °C to 90 °C, and then from 90 °C to 27 °C.

By indicating with $SD_{max}$ the maximum value of *SD* and with $SD_{final}$ the final value of *SD*, we can define a spectral thermal resilience parameter by means of the following formula:

(3) $$SR=\frac{SD_{max}-SD_{final}}{SD_{max}}$$

For our systems, the value of the spectral thermal resilience is 0.19 for the aqueous solution of Lysozyme while it is 0.61 for the Lysozyme aqueous solution in presence of PEG 400. Such a result confirms what it emerges from the thermal restraint parameter i.e., that the addition of PEG 400 to aqueous solution of Lysozyme increases the system capability to afford temperature stress, as well as indicates a higher capability to recover the original properties [56,57,58,59,60].

## 3. Experimental Setup and Sample Preparation

Lysozyme, PEG with M_w_ of 400 corresponding to values of the polymerization degree *m* = 8, and distilled water were purchased from Aldrich-Chemie. FT-IR data were collected both increasing and decreasing temperature in the 20 °C ÷ 90 °C range.

The investigated concentration values are expressed in percentage by weight; for the binary system, 70% of Lysozyme and 30% of water, while for the ternary system, 70% of Lysozyme and 30% of (50% water + 50% PEG 400).

To collect the IR spectra a FTIR-Vertex 70 V (Bruker Optics, Germany) spectrometer by Bruker Optics using a Platinum diamond ATR has been employed. Previously, to interpretate the spectra, some data preprocessing has been applied by means of Bruker OPUS/Mentor software (Bruker Optics, Germany) and MATLAB environment. More precisely, in order to reduce the variations between spectra due to baseline shift, a baseline treatment has been performed; to decrease the instrumental noise, a smoothing treatment has been applied; and, finally, in order to correct the path length variation and to decrease the variations among each measurement, a normalization of spectra has been taken into account. To confirm the caliber and accuracy of spectral data, a performance qualification (PQ) test using the fully automated validation program of OPUS 7.5 software (furnished by Bruker Optics) was carried out. The ATR diamond crystal’s surface was in direct contact with the deparaffinized breast tissue slices (2 mm × 2 mm), and the mid-IR range of 4000 cm^−1^ to 400 cm^−1^ was transmitted to and from the ATR accessory. To acquire a suitable signal-to-noise ratio, spectra were created at a spatial resolution of 0.4 cm^−1^, and an average of 48 scans was collected [61,62,63]. Each sample’s background spectrum was collected before it was scanned, and the software used this spectrum to routinely remove ambient effects. More precisely, we have performed 48 scans for each of the 13 measurements by means of the Vertex 70v Bruker IR spectrometer; thanks to the temperature sensor set in the instrument and by means of OPUS software we have been able to change the values of temperature from 27 °C to 90 °C and from 90 °C to 27 °C. In particular, at first, we increase the temperature from the first spectrum at 27 °C, then the second at 30 °C, the third at 50 °C, the forth at 65 °C, the fifth at 75 °C, the sixth at 80 °C and the seventh at 90 °C, then, we decrease the values temperature, obtaining the eighth at 80 °C, the nineth at 75 °C, the tenth at 65 °C, the eleventh at 65 °C, the twelfth at 50 °C and the thirteenth, the last, at 27 °C.

## 4. Conclusions

IR absorption data collected on Lysozyme aqueous solutions also in the presence of PEG 400 as a function of temperature have been analyzed by evaluating the SD.

Concerning the increasing temperature ramp, to extract quantitative information on the system thermal response, the SD values as a function of temperature have been fitted by means of sigmoid curve which allowed to extract the sigmoid amplitude, which is connected to the value of thermal restraint, the sigmoid steepness, and $T_{0}$ the relaxational temperature. Such an analysis shows that for the binary system, that value of amplitude is higher, whereas the value of temperature is lower for the binary system in respect to the ternary system. The obtained result shows that the addition of PEG 400 to Lysozyme, gives rise to smaller changes in the features of the registered spectra suggesting that the aqueous solution of Lysozyme in the presence of PEG 400 are more resistant to the temperature changes. The obtained results are in line with previous experiments performed by Raman scattering on Lysozyme/water/bioprotectant systems that have evidenced a similar sigmoid behavior for the protein *Amide I* center frequencies.

Furthermore, we have introduced a spectral thermal resilience parameter which has been connected to the capability to recover the original spectral features after a thermal stress cycle. The value obtained for the spectral thermal resilience parameter indicates that the addition of PEG 400 to aqueous solution of Lysozyme increases the system capability to afford temperature stress, as well as indicating a higher capability to recover the original properties.

## Figures and Tables

**Table 1:** Values of Amplitude (A), Steepness (B) and Temperature ($T_{0})$ extracted from Equation (2) for the two investigated systems.

	Amplitude	Steepness	Temperature (°C)
Lysozyme/H_2_O	0.975	−0.01283	55.32
Lysozyme/H_2_O/PEG 400	0.697	−0.01577	58.55

## Data Availability

The data presented in this study are available on request from the corresponding author.

## Sample Availability

Samples of the compounds are not available from the authors.
